# Formulation A in the prevention of rheumatoid arthritis: a study protocol for a multicenter randomized controlled trial

**DOI:** 10.3389/fmed.2025.1623474

**Published:** 2025-09-12

**Authors:** Kaixin Gao, Yu Wen, Yihang Yu, Jianhong Peng, Weidong Liang, Zhengdong Shen, Lei Zhang, Yongliang Chu, Liyan Mei, Haifang Du, Xiumin Chen, Maojie Wang, Zehuai Wen, Aikaterini Chatzidionysiou, Per-Johan Jakobsson, Runyue Huang

**Affiliations:** ^1^State Key Laboratory of Traditional Chinese Medicine Syndrome, The Second Affiliated Hospital of Guangzhou University of Chinese Medicine (Guangdong Provincial Hospital of Chinese Medicine), The Second Clinical Medical College of Guangzhou University of Chinese Medicine, Guangzhou, China; ^2^Dongguan Hospital of Traditional Chinese Medicine, Dongguan, Guangdong, China; ^3^Guangdong Provincial Hospital of Chinese Medicine Zhuhai, Zhuhai, Guangdong, China; ^4^State Key Laboratory of Dampness Syndrome of Chinese Medicine, The Second Affiliated Hospital of Guangzhou University of Chinese Medicine (Guangdong Provincial Hospital of Chinese Medicine), Guangzhou, China; ^5^Guangdong Provincial Key Laboratory of Clinical Research on Traditional Chinese Medicine Syndrome, Guangzhou, China; ^6^The key unit of methodology in clinical research, The Second Affiliated Hospital of Guangzhou University of Chinese Medicine, Guangzhou, China; ^7^Department of Medicine, Division of Rheumatology& Karolinska University Hospital at Solna, Rheumatology Clinic, Karolinska Institutet, Stockholm, Sweden

**Keywords:** rheumatoid arthritis, prevention, traditional Chinese medicine, formulation A, randomized controlled trial, protocol

## Abstract

**Background:**

Interventions to delay or prevent the onset of rheumatoid arthritis (RA) have attracted much attention in recent years. Researchers are now exploring various prevention strategies but no unified consensus was reached. A traditional Chinese medicine (TCM) prescription, Formulation A (FA), may be potential to reduce the risk of RA development.

**Methods:**

This is a multicenter, double-blind, placebo-controlled, randomized clinical trial. We will enroll 72 participants who are positive for anti-cyclic citrullinated peptide (anti-CCP) antibody but without synovitis. Participants will be randomly assigned to either FA or placebo for 52 weeks. All will then undergo 52 weeks of observation. The primary outcome is the time interval between the study entry and RA diagnosis. The secondary outcomes include the proportions of RA diagnosis, anti-CCP antibody, rheumatoid factor (RF), the high-sensitivity C-reactive protein (hs-CRP), the erythrocyte sedimentation rate (ESR), the 28-tender joint count (TJC28), the 28-swollen joint count (SJC28), morning stiffness time, the visual analog scale (VAS), Patient’s Global Assessment (PtGA), Physician’s Global Assessment (PGA), Health Assessment Questionnaire (HAQ), Generic Quality of Life Inventory-74 (GQOLI-74) scores, and ultrasound images of joints.

**Discussion:**

This study attempts to test the feasibility of a clinical trial using FA in RA prevention. It will lay the foundation for future research and ensure validity.

## 1 Introduction

Rheumatoid arthritis (RA) is one of the most common autoimmune diseases worldwide which can lead to severe joint damage by causing inflammation of synovium. Extra-articular manifestations are also common, and overall, the disease causes high social and economic losses (1).

Although awareness of early diagnosis, treat-to-target strategies, and the latest biologic agents have led us into a new era of RA treatment, we should continue to focus on disease prevention. Therefore, researchers have proposed recommendations on preclinical RA (pre-RA) and clinically suspect arthralgia (CSA) to help identify individuals at risk of developing RA (2, 3).

However, finding the most appropriate drugs and therapies for high-risk individuals is not easy. The causes of the disease are multiple and complex (4). And the concepts and descriptions of the at-risk stage of RA are still evolving (2). Both genetic and environmental factors contribute strongly to the development of RA, leading to epigenetic and posttranslational modifications, which in turn trigger autoimmune responses through the activation of immune cells and the formation of autoantibodies (5, 6). The at-risk stage can be recognized as the period with some clinical manifestations until the onset of clinical arthritis. Years before the onset of RA, anti-cyclic citrullinated peptide (anti-CCP) antibodies are frequently found (7). Musculoskeletal manifestations, such as arthralgia, bone loss and fatigue, are also common (8). When these at-risk individuals are also ever smokers and carriers of genetic risk alleles, the risk of developing RA increases more than 20-fold (9). These existing at-risk cohorts have been recently summarized (10). They could be the target of the current prevention trial (11).

Since the mechanisms of RA pathology involve multiple immune cells, effector cells, and stromal cells and many different intracellular signaling pathways, a drug or a combination of active compounds with a wide range of actions might be preferable. Can TCM be useful and provide effective solutions for RA prevention? Previous studies have shown that TCM can lead to a decrease in the levels of rheumatoid factor (RF) and anti-CCP (12), reduce bone damage (13) and achieve a strong therapeutic effect (14), which could be a potentially effective choice for RA prevention.

TCM has been applicated in clinical settings in China for millennia. Formulation A (FA), known as Yiqi-Qushi-Fangbi-Fang (YQFF) in the Chinese nomenclature, has been selected for its role in RA prevention in clinical practice. FA consists of eight different common Chinese herbs, as shown in Table 1 (11). It shows the potential to alleviate joint symptoms, such as pain, numbness, stiffness, distension, swelling, limb flexion and extension, so as to decrease the risk of RA.

**TABLE 1:** Components and their names in formulation A.

Chinese	Pinyin	Scientific name	Latin name	Officinal parts	Doses
薏苡仁	Yiyiren	Coix lacryma-jobi var. ma-yuen (Rom.Caill.) Stapf	Semen Coicis	Seed	30 g
当归	Danggui	Angelica sinensis (Oliv.) Diels	Radix Angelicae Sinensis	Root	10 g
白芍	Baishao	Paeonia lactiflora Pall.	Radix Paeoniae Alba	Root	10 g
麻黄	Mahuang	Ephedra sinica Stapf	Herba Ephedrae	Stem	3 g
桂枝	Guizhi	Cinnamomum cassia Presl.	Ramulus Cinnamomi	Branch	10 g
黄芪	Huangqi	Astragalus mongholicus Bunge	Radix Astragali seu Hedysari	Root	30 g
苍术	Cangzhu	Atractylodes lancea (Thunb.) DC.	Rhizoma Atractylodis	Root, stem	15 g
炙甘草	Zhigancao	Glycyrrhiza uralensis Fisch.	Radix Glycyrrhizae	Root, stem	15 g

In fact, FA represents a synthesis and innovative adaptation of two distinguished TCM formulas, Huangqi-Guizhi-Wuwu-Tang (HGWT) and Yiyiren-Tang (YYRT).

The application of HGWT for the treatment of RA in China has a long history. Modern pharmacological studies have shown that monoterpenoids, flavonoids, organic acids, triterpenes, amino acids, gingerols, alkaloids, and glycosides are the main chemical components in HGWT (15–17). It has anti-RA effects by targeting multiple pathways (18), such as regulating neurotransmitters (19); adjusting macrophages and the levels of inflammatory cytokines (20); decreasing the mRNA and protein levels of p-NF-κB and IL-17 (21); reducing inflammation in fibroblast-like synoviocytes (22); and synergistically targeting the cAMP, PI3K/Akt, and HIF-1α pathways (23). In addition, HGWT is widely used in treating neurological disorders, such as neuropathic pain (24), peripheral neuropathy (25–27), peripheral neurotoxicity (28, 29), and stroke rehabilitation (24).

YYRT has adjuvant therapeutic value for RA through diverse anti-inflammatory and immunomodulatory effects. Coix seeds, the main component of YYRT, contain coix seed ester and polysaccharide components, which can exert anti-RA effects by reducing the levels of proinflammatory cytokines (IL-1β, TNF-α, IL-6, and MCP-1), alleviating oxidative stress (30), and alleviating synovial angiogenesis by suppressing the HIF-1α/VEGF-A signaling pathway (31). A compound study revealed that YYRT might improve the treatment effect against RA, particularly the cold-dampness obstruction type (32).

FA, the combination of HGWT and YYRT, broadens the application spectrum of TCM therapeutic approaches. Its ingredients contain anti-inflammatory and immune-regulating components, making it suitable for the high-risk stage of RA. Also, it has a more palatable taste, which can significantly improve patients’ medication adherence. However, there is a lack of high-quality evidence-based studies on FA. Thus, we outline an ongoing multicenter clinical trial to explore its role in RA prevention.

## 2 Methods and analysis

### 2.1 Objectives

This study aims to evaluate the feasibility of a multicenter clinical trial of FA for RA prevention.

### 2.2 Study design

This is a study protocol of a multicenter, double-blind, randomized controlled trial (RCT). Six centers are involved in this RCT, including the Guangdong Provincial Hospital of Chinese Medicine (GPHCM), the Guangdong Provincial Hospital of Chinese Medicine Zhuhai, the Dongguan Hospital of Guangzhou University of Traditional Chinese Medicine, The Second Affiliated Traditional Chinese Medicine Hospital of Guizhou University of Traditional Chinese Medicine, the Liaocheng Traditional Chinese Medicine Hospital, and the Inner Mongolia Hospital of Traditional Chinese Medicine. The protocol was approved by the GPHCM ethics committees (BF2021-159-01) on September 8, 2021, and study was registered in the Chinese Clinical Trial Registry on October 1, 2021^1^. We started recruitment in March 2022 and plan to finish in 2026.

### 2.3 Eligibility criteria

#### 2.3.1 Inclusion criteria

Participants must:

(1)    be aged between 18 and 75 years (inclusive) at the time of initial screening.
(2)    Test positive for anti-CCP, regardless of RF status.
(3)    Be present with or without arthralgia that may be accompanied by joint discomfort, such as pain or stiffness, but not synovitis.
(4)    Not meet the 2010 American College of Rheumatology/European League Against Rheumatism classification criteria for RA as confirmed by a certified rheumatologist.
(5)    Have no prior exposure to any DMARDs.
(6)    Eligible individuals must agree to participate in the trial and sign an informed consent form.

#### 2.3.2 Exclusion criteria

Any of the following circumstances must be excluded:

(1)    A history of other rheumatic autoimmune diseases, such as Sjögren syndrome or systemic lupus erythematosus; acute or chronic infections, including hepatitis B or hepatitis C; evidence of active, latent or inadequately treated Mycobacterium tuberculosis infection; or a history of cancer.
(2)    Severe diseases of the brain, lungs, liver, kidneys, cardiovascular system, or hematopoietic system or diabetes.
(3)    Pregnant women, nursing mothers, or individuals with psychiatric disorders.
(4)    A hemoglobin level of less than 90 g/L, a white blood cell count of less than 3.0 × 10^9^/L, or a platelet count of less than 100 × 10^9^/L.
(5)    The estimated glomerular filtration rate (eGFR) of 40 mL/min or less (calculated via the method of Cockcroft and Gault).
(6)    The aspartate aminotransferase (AST) or alanine aminotransferase (ALT) levels greater than 1.5 times the upper limit of the normal range.
(7)    Active gastroduodenal ulcers or gastritis caused by long-term use of nonsteroidal anti-inflammatory drugs (NSAIDs).
(8)    Hypersensitivity to the trial medication.
(9)    Synovitis detected by ultrasound prior to the inclusion.
(10)    Previous use of glucocorticoids, regardless of whether oral or topical treatment was used.
(11)    Participation in other clinical trials within 4 weeks prior to screening.

#### 2.3.3 Recruitment

There are four main approaches for recruitment:

(1)    Outpatient clinics at the six centers: Physicians conduct an initial assessment of patients arriving at their clinics.
(2)    Population health screening: a blood test for the anti-CCP antibody is included in the health screening service to identify people who are anti-CCP positive.
(3)    News media and advertisement: Residents can volunteer for screening through newspapers, advertisements, WeChat public accounts, telephone counseling, video media, etc.
(4)    Other hospitals: eligible patients will be recommended for this study.

After preliminary screening, individuals who are considered potential participants in this trial will undergo a more detailed assessment according to the inclusion and exclusion criteria. If the eligibility criteria are met, informed consent (including additional consent provisions for the collection and use of participant data and biological specimens in ancillary studies) will be provided and explained by the clinician and should be signed by the participant.

### 2.4 Intervention

#### 2.4.1 Intervention granules

The participants will be randomly assigned to take FA granules or placebo granules orally twice daily after meals for a total of 52 weeks. The outer packaging will be recycled and brought back to the researcher at each point.

#### 2.4.2 Granule production procedures

**FIGURE 1:**
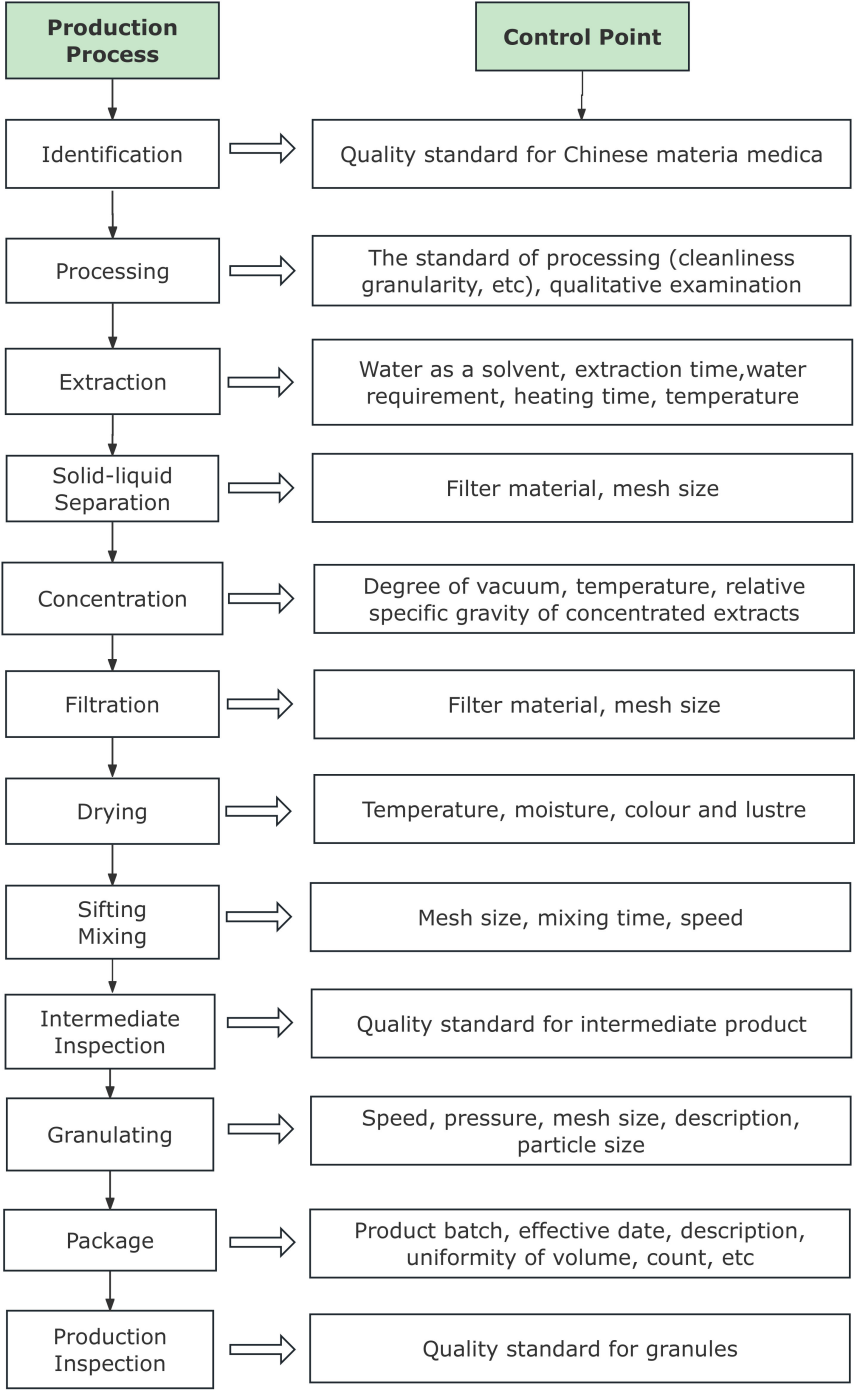
Production process of the granules.

FA is made from multiple plants (Table 1). The granule-making procedure is shown in Figure 1.

The placebo was invented and is produced by Tianjiang Pharmaceutical Co., Ltd. It consists of simple ingredients, such as raw materials and excipients, including maltodextrin bitterant and coloring matter (lemon pigment, sunset yellow pigment and caramel pigment). All the materials are of food grade, and no active ingredient is included, which excludes any notable adverse reactions or threats to the health of human beings. FA and the placebo are identical in appearance, including dosage form, aluminum-plastic packaging, and labeling. All participants receive standardized administration instructions stating “The medication has a slightly sweet taste with mild bitterness” to minimize expectation bias.

#### 2.4.3 Quality control of granule production

##### 2.4.3.1 Stability test of FA Granules

For multiple batches of FA, a similarity of more than 96% must be detected by ultra-performance liquid chromatography (UPLC), indicating that the ingredients of FA are stable and controllable. The procedure for UPLC fingerprinting of FA granule ingredients between batches is performed according to a published protocol (33, 34).

##### 2.4.3.2 Fingerprint chromatograms of FA granules

The fingerprint chromatogram of FA is obtained via high-performance liquid chromatography (HPLC) for quality control. The identification of FA compounds relies on their retention times and UV spectra compared with those of standards.

##### 2.4.3.3 Similarity analysis

This manual assessment method is carried out to determine the similarity between the placebo and FA. Twenty individuals of different ages and sexes who have neither visual (e.g., color blindness or color deficiency) nor olfactory problems are asked to independently evaluate the samples prepared versus placebo. Compared with the FA granules, the placebo granules that are completely different in color, appearance and smell are scored 0 points. Those that are completely identical are awarded 100 points. The scores of colors, appearance and smell are collected to calculate the average score. Considering the significant influence of the odor and color of the placebo, the composite score of the three variables is calculated based on the following weighting: 20% for appearance, 40% for color and 40% for odor. If the composite score is in the range of 80–100 points, this indicates a relatively high similarity with FA, and the sample can be used as a placebo for FA.

#### 2.4.4 Discontinuation criteria, adherence and concomitant therapy

Long-term medications for the treatment of underlying diseases, such as high blood pressure, are allowed. Other symptomatic treatments, such as NSAIDs, may be used if the investigators judge that they will not interfere with the assessment of the study drug. We strictly prohibit glucocorticoids, either oral or topical. All concomitant medications should be recorded in the case report form (CRF).

The trial intervention should be stopped in the following circumstances: if serious adverse events (SAEs) occur; if the patient’s condition deteriorates drastically during the trial and it cannot be ruled out that the change is related to the trial, the study should be terminated, and the necessary treatment measures should be taken for the patient; and if patients withdraw their consent.

The duration of the intervention phase and subsequent follow-up in this study are notably extensive, particularly for participants who are asymptomatic. To improve medication adherence, our approaches encompass the following aspects:

(1)    Relationship enhancement: An equitable doctor–patient rapport will be established to ensure that patients are treated with dignity and support and have an increased sense of engagement and commitment.
(2)    Incentive policy: Full reimbursement will be provided for all medication and testing expenses of the participants and will be complemented by a modest financial compensation, such as transportation allowances and minor stipends. Nevertheless, ensuring that these measures do not appear to be a means of inducing participation is crucial.
(3)    Technological assistance: Electronic devices and applications can be utilized to implement automated medication reminders, thereby promoting adherence to treatment regimens.
(4)    Customized management: Acknowledging the diverse challenges faced by individual participants, such as work schedule conflicts, adaptable appointment schedules will be offered. For patients with limited education or advanced age, simplified educational materials, such as visual aids, can be designed. With respect to patients from different cultural or religious backgrounds, we will accommodate their diversity and offer patient-centered personalized services.
(5)    Collaborative teamwork: The research team will convene for regular internal meetings to share effective strategies and address encountered obstacles, ensuring that all members have access to current information and support for enhancing patient management and communication skills.

### 2.5 Outcomes

#### 2.5.1 Demographic characteristics

Age, gender, height, weight, body mass index (BMI), current medication use, comorbidities, and family history.

#### 2.5.2 The primary outcome

The primary outcome is the time to the occurrence of RA. It is defined as the time interval from inclusion to the occurrence of RA. RA occurrence refers to the confirmation of arthritis or synovitis by ultrasound and meeting the 2010 ACR/EULAR RA classification criteria.

#### 2.5.3 Secondary outcomes

The secondary outcomes include the following:

(1)    Proportions of participants who develop RA at different observation time points.
(2)    Autoantibodies: a reduction in anti-CCP or RF titer.
(3)    Biochemical tests: the high-sensitivity C-reactive protein (hs-CRP) level and the erythrocyte sedimentation rate (ESR).
(4)    Joint symptoms and signs: the 28-tender joint count (TJC28), 28-swollen joint count (SJC28), visual analog scale (VAS) score, morning stiffness, and other complaints, such as pain and numbness.
(5)    Systemic symptoms and signs: the Patient’s Global Assessment (PtGA) score, Physician’s Global Assessment (PGA) score, Health Assessment Questionnaire (HAQ) score, and Disease Activity Score in 28 joints-C-reactive Protein (DAS28-CRP).
(6)    Generic Quality of Life Inventory-74 (GQOLI-74) score.
(7)    Doppler ultrasound of both hands, feet and other affected joints: (A) Mandatory joints: the radiocarpal and midcarpal joints, the distal radioulnar joint, and the 6th compartment; the metacarpophalangeal (MCP) joints in the dorsal position in the second to fifth fingers bilaterally; the proximal interphalangeal (PIP) joints and flexor tendons in the volar position in the second to fifth fingers bilaterally; and the metatarsophalangeal (MTP) joints in the dorsal position in the 2nd to 5th toes in the feet bilaterally. (B) Symptomatic joints: if patients report symptoms in joints other than those described above, the sono-rheumatologist will scan these symptomatic joints at the same time to determine whether there are any sonographic changes associated with synovitis, tenosynovitis, tendinitis, enthesitis or bursitis in these areas (Supplementary Appendix 1).
(8)    Digital radiography (DR) of the hands and feet.

#### 2.5.4 Safety assessment

Safety assessment includes complete blood count (CBC), urinalysis (UA), liver function tests (LFTs) (AST and ALT), renal function tests (RFTs) (creatinine and urea), electrocardiogram (ECG), chest X-ray, and adverse events (AEs),

### 2.6 Participant timeline

Fifty-two weeks of medical intervention will be followed by another 52 weeks of observation, for a total of 104 weeks. The time points for the measurements are as follows: screening (0 days), 4 ± 3 days, 12 ± 5 days, 24 ± 5 days, 52 ± 5 days, 78 ± 5 days and 104 ± 5 days. During the trial and follow-up, participants will be observed and evaluated via clinical and/or imaging examinations.

### 2.7 Sample size

As this trial primarily assessed feasibility on RA prevention by TCM, sample size was not strictly predetermined. We initially allocated 30 participants per group with an additional 20% potential dropouts, resulting in a total of 72 recruitment. Nevertheless, we provide a formal calculation for reference purposes.

According to previous study (35), we assumed that RA incidence would be 20.0% in the FA group and 37.5% in the placebo group. This allocation ensured an power of over 80%. Calculation was in accordance with the formula of Chow (36) as follow:


$$\text{N}=\frac{{\left({\text{Z}}_{\alpha }+{\text{Z}}_{\beta }\right)}^{2}\,\left[{\text{P}}_{0}\,\left(1-{\text{P}}_{0}\right)+{\text{P}}_{1}\,\left(1-{\text{P}}_{1}\right)\right]}{{\left(\varepsilon +\delta \right)}^{2}}$$

Note: *N* is the sample size of each group. P_0_ and P_1_ are the RA incidence in the FA group and placebo group, respectively. For a two-sided test, α = 0.05, β = 0.2, 1−β = 0.8, Z_α_ = 1.96, Z_β_ = 0.84. The superiority margin δ = 0.15. And ε is the practical margin.

### 2.8 Randomization and blinding

The randomization will be performed via the block randomization method by using the PROC PLAN procedure of SAS V9.4, which is administered by the Key Unit of Methodology in Clinical Research (KUMCR) at the GPHCM. According to the sample size estimate, 72 patients will be included in this study and randomly divided into two groups at a 1:1 ratio. To avoid wasting random numbers, two extra random numbers were added, and the adjusted random number is 74. Once the sample size reaches 72, patient enrolment will cease, even though there might still be unused random numbers at that point. After informed consent is obtained from participants who meet the eligibility criteria, clinicians can access the online random allocation system to procure the allocation results and immediately implement the intervention measures. When multiple patients are randomly assigned on the same day, the application procedure should be performed one by one. The patients, clinicians and investigators of this trial, including study assistants and statisticians, are blinded to the assignment. Unblinding is permitted in an emergency if a SAE occurs or if the intervention must be known for rescue.

### 2.9 Data collection, management, and analysis

#### 2.9.1 Data collection and management

The identification, registration and subsequent follow-up are performed by trained clinicians via questionnaires, physical examinations and laboratory tests. All the investigators and study assistants will attend a training workshop to ensure that they adhere to the study protocol. In addition, a standard operating procedure (SOP) will be prepared and sent to the entire team. Clinical data will be recorded in the CRF by the investigators and will be monitored and reviewed regularly by the data manager and inspectors from the GPHCM Scientific Research Department. The data will be entered by two different operators independently. Archiving and locking occur after two independently entered data points are compared and verified to be error-free. Missing data, suspect data and conflicting data are re-evaluated.

#### 2.9.2 Statistical methods

Data analysis will be conducted by statisticians who are independent of the research team. All analyses are performed via the SPSS software (IBM SPSS Inc., Armonk, NY, USA). An intent-to-treat analysis will be carried out for participants who receive treatment at least once. Missing data will be imputed in accordance with the principle of multiple imputations. Continuous variables will be imputed using linear regression models, while categorical variables will be imputed using logistic regression models. Multiple imputation was performed with a user-defined number of MCMC (Markov Chain Monte Carlo) iterations. Per-protocol analysis is limited to participants who strictly adhere to the protocol and complete the trial. All the statistical tests are 2-sided, and differences are considered statistically significant at *P* values less than 0.05.

The safety dataset will also include all patients who received ≥1 dose of the intervention. The primary outcomes will be compared between the two groups via the log-rank test and the Kaplan–Meier method, and superiority will be considered with a 95% confidence interval. The secondary outcomes will be presented as frequencies, means, standard deviations (SDs), medians and ranges. To determine treatment effects, changes in the above outcomes from baseline will be analyzed via ANOVA. Group effects and influencing factors will be analyzed via analysis of covariance or a logistic regression model with covariates including sex, age, concomitant medication and measurements at baseline. Subgroup analyses will not be performed to avoid insufficient statistical power because of the small sample size. Safety will be evaluated through tabulations of AEs and coded based on the Chinese version of the Medical Dictionary for Regulatory Activities (MedDRA). Safety data will be presented with descriptive statistics at baseline, 52 weeks and 104 weeks for each group.

#### 2.9.3 Monitoring

Any adverse events (AEs) that occur during the study period will be reported to the investigators and recorded in the CRFs. In addition to participant self-reports, abnormal laboratory findings, including routine blood tests, urine tests, liver function tests, kidney function tests and pathological electrocardiograms with clinical significance, will be recorded as AEs. AE details, including the time of occurrence, severity, causality with the intervention, actions taken, and AE outcomes, will be observed and assessed. Causalities between AEs and the FA granules will be assessed according to the WHO Uppsala Monitoring Centre System for Standardized Case Causality Assessment^2^. Any severe AEs or serious adverse drug reactions will be addressed promptly to ensure patient safety and will be reported to the principal investigator and the hospital ethics committee in a timely manner.

**FIGURE 2:**
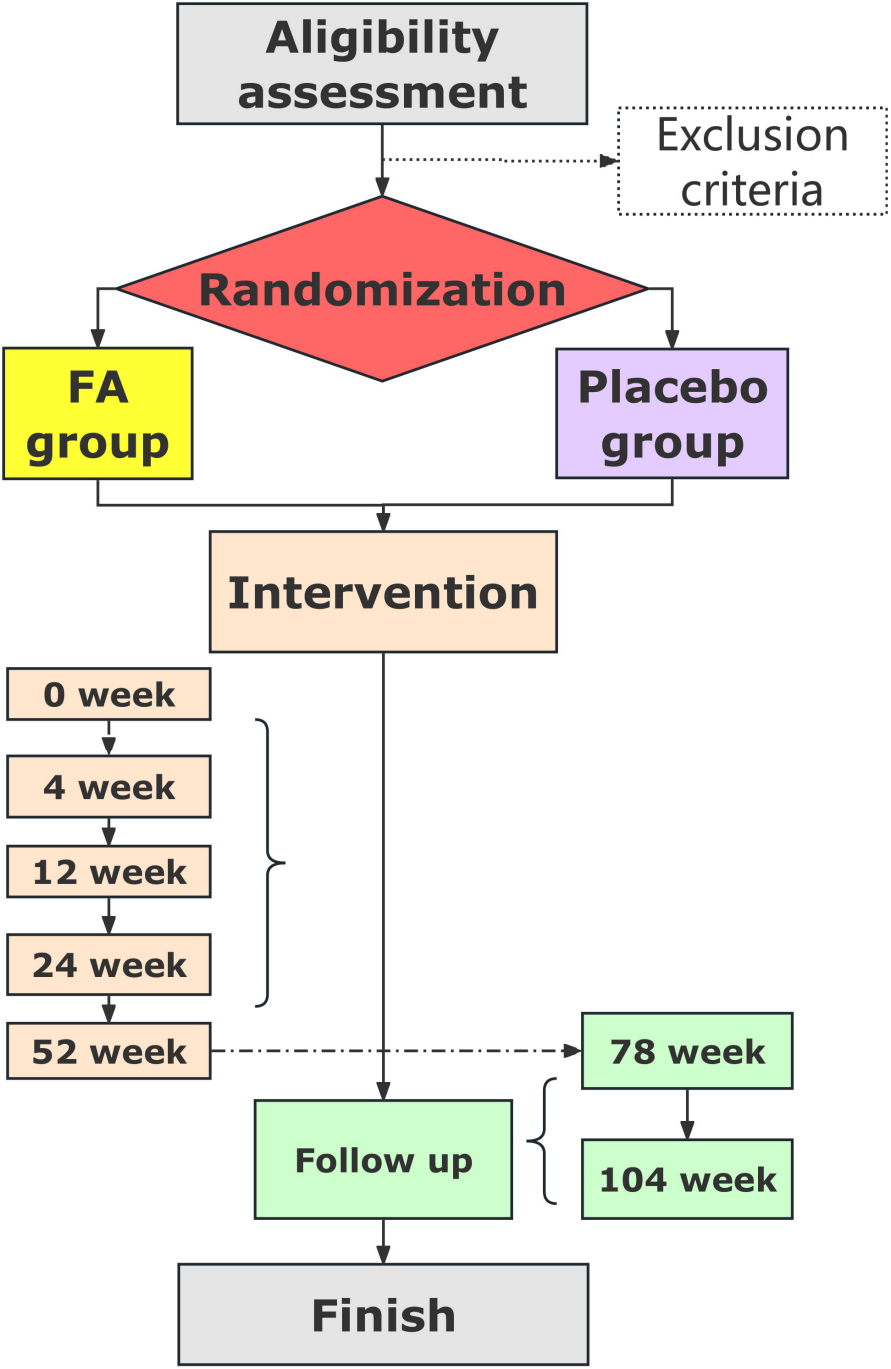
Trial flowchart.

The trial flowchart is shown in Figure 2, and the schedules and measurements for the participants are listed in Table 2.

**TABLE 2:** Time schedule and measurements.

Assessment	Visit 1	Visit 2	Visit 3	Visit 4	Visit 5	Visit 6	Visit 7
	Enrollment	4^th^ w	12^th^ w	24^th^ w	52^th^ w	78^th^ w	104^th^ w
Demographic data	×						
Family history	×						
Medical history	×						
Physical examination	×	×	×	×	×	×	×
Vital signs	×	×	×	×	×	×	×
Disease activity	×	×	×	×	×	×	×
Morning stiffness	×	×	×	×	×	×	×
TJC28	×	×	×	×	×	×	×
SJC28	×	×	×	×	×	×	×
HAQ	×	×	×	×	×	×	×
VAS	×	×	×	×	×	×	×
PtGA	×	×	×	×	×	×	×
PGA	×	×	×	×	×	×	×
GQOLI-74	×			×	×	×	×
anti-CCP	×		×	×	×	×	×
RF	×	×	×	×	×	×	×
hs-CRP	×	×	×	×	×	×	×
ESR	×	×	×	×	×	×	×
DAS28-CRP	×	×	×	×	×	×	×
CBC	×	×	×	×	×	×	×
UA	×	×	×	×	×	×	×
LFTs	×	×	×	×	×	×	×
RFTs	×	×	×	×	×	×	×
ECG	×				×	×	×
Chest radiography	×				×	×	×
DR of the joints	×				×	×	×
Ultrasound	×		×	×	×	×	×
Concomitant medication	< — — — — — — — — — — — — — — — — — — — — — — — — — — >						
Adverse events	< — — — — — — — — — — — — — — — — — — — — — — — — — — >						
Concomitant medications	< — — — — — — — — — — — — — — — — — — — — — — — — — — >						

TJC28, 28-tender joint count; SJC, 28-swollen joint count; HAQ, health assessment questionnaire; VAS, visual analog scale; PtGA, patient’s global assessment; PGA, physician’s global assessment; GQOLI-74, generic quality of life inventory-74; anti-CCP, anti-cyclic citrullinated peptide; RF, rheumatoid factor; hs-CRP, high-sensitivity C-reactive protein; ESR, erythrocyte sedimentation rate; DAS28-CRP, disease activity score in 28 joints-C-reactive protein; CBC, complete blood count; UA, urinalysis; LFTs, liver function tests; RFTs, renal function tests; ECG, electrocardiogram; DR, digital radiography.

## 3 Biobank

Biological samples, including blood, urine and stool samples, will be collected from study participants at each observation time point for future studies to investigate biomarkers for RA prevention.

## 4 Discussion

Although previous studies have tested the use of DMARDs to delay the onset of RA (37, 38), there is also a growing need for clinical evidence for TCM.

However, this trial has several potential limitations. It is a small-scale pilot trial, and therefore, the results may not be conclusive or sufficiently generalizable. Nevertheless, any significant differences or trends in the efficacy of FA at the endpoints of this trial will provide seminal evidence for the search for RA prevention, and even neutral results will be important revelations to the community. RA is a serious global rheumatic disease that affects many people both physically and mentally.

With the rapid advances in science and medicine in recent years, remission is no longer the only goal to meet the needs of RA patients. Medical care and management strategies for at-risk individuals are becoming a priority for clinicians and researchers. This trial can provide high-quality data and hopefully suggest a new therapy to prevent rheumatoid arthritis.

## 5 Study status

This study is ongoing and currently in the patient recruitment phase.

## Footnotes

1 ChiCTR2100051741, URL:https://www.chictr.org.cn/showproj.html?proj=133579

2
https://www.who-umc.org/media/164200/who-umc-causality-assessment_new-logo.pdf
