# Supplementary material for: Formulation A in the prevention of rheumatoid arthritis: a study protocol for a multicenter randomized controlled trial
Source: Front Med (Lausanne). 2025 Sep 12;12:1623474. doi: 10.3389/fmed.2025.1623474 (PMC12463902; doi:10.3389/fmed.2025.1623474)
Supplement: Supplementary file 1 [file Data_Sheet_1.PDF]

The ULTRASOUND examination protocol:

a) mandatory joints

- Bilateral wrist in 4 positions
    - Longitudinal central position to scan radiocarpal and midcarpal joints (landmark: Radius, Lunate, Capitate, the proximal part of metacarpal bones and the 4:th compartment should be visualized).
    - Longitudinal radial position to scan radiocarpal and midcarpal joints (landmark: Radius, Scaphoid, Trapezoid and the 2:d compartment should be visualized).
    - Longitudinal ulnar position to scan distal radio-ulnar joint (Landmark: Ulnar head, Triquetrum, Hamate and the proximal part of metacarpal bones should be visualized)
    - Longitudinal lateral position to scan the 6:th compartment

- Metacarpophalangeal (MCP) joints in dorsal position in the second to fifth fingers bilaterally

- Proximal interphalangeal (PIP) joints and flexor tendons in volar position in the second to fifth fingers bilaterally

- Metatarsophalangeal (MTP) joints in dorsal position in the 2 to 5 in the feet bilaterally

b) symptomatic joints

if the patients report symptoms from joints other than the above, the sono-rheumatologist scans these symptomatic joints at the same time in order to see if there are any sonographic changes for synovitis, tenosynovitis tendinitis/enthesitis or bursitis in these areas.

This is the protocol that use in rheumatology dept for all patients with suspected arthritis.
